# The Bidirectional Association Between Cognitive Function and Gait Speed in Chinese Older Adults: Longitudinal Observational Study

**DOI:** 10.2196/44274

**Published:** 2023-03-14

**Authors:** Haibin Li, Jiajia Zhang, Xinye Zou, Xiuqin Jia, Deqiang Zheng, Xiuhua Guo, Wuxiang Xie, Qi Yang

**Affiliations:** 1 Department of Cardiac Surgery Beijing Chaoyang Hospital Capital Medical University Beijing China; 2 Heart Center and Beijing Key Laboratory of Hypertension Beijing Chaoyang Hospital Capital Medical University Beijing China; 3 Department of Radiology Beijing Chaoyang Hospital Capital Medical University Beijing China; 4 Department of Education University of Cambridge Cambridge United Kingdom; 5 Department of Epidemiology and Health Statistics School of Public Health Capital Medical University Beijing China; 6 Peking University Clinical Research Institute Peking University First Hospital Beijing China; 7 Beijing Laboratory for Cardiovascular Precision Medicine Beijing China; 8 Key Laboratory of Medical Engineering for Cardiovascular Disease Ministry of Education Beijing China

**Keywords:** aging, cognitive function, gait speed, cross-lagged panel models, longitudinal study

## Abstract

**Background:**

Cognitive and gait speed decline are common conditions in older adults and are often associated with future adverse consequences. Although an association between cognitive function and gait speed has been demonstrated, its temporal sequence remains unclear, especially in older Chinese adults. Clarifying this could help identify interventions to improve public health in older adults.

**Objective:**

This study aims to examine the longitudinal reciprocal association between gait speed and cognitive function and the possible temporal sequence of changes in both factors in a national longitudinal cohort.

**Methods:**

Data were derived from 2 waves (2011 baseline and 2015 follow-up) of the China Health and Retirement Longitudinal Study (CHARLS). Participants 60 years or older, without dementia or Parkinson disease at baseline, and with completed data on gait speed and cognition at both baseline and follow-up were included. Usual gait speed was measured over two 2.5-m walks. Mental intactness and episodic memory were used to assess global cognitive function. Cross-lagged panel models and linear mixed-effects models were used to examine the association between cognition and gait speed over time. Standardized coefficients were reported.

**Results:**

A total of 3009 participants (mean age 66.4 years, SD 5.4 years; 1422/3009, 47.26%, female participants) were eligible for inclusion in our analyses. Cross-lagged panel analyses revealed that after accounting for baseline gait speed, cognition, and potential confounders, baseline global cognition (β=.117, 95% CI 0.082-0.152; *P*<.001), mental intactness (β=.082, 95% CI 0.047-0.118; *P*<.001), and episodic memory (β=.102, 95% CI 0.067-0.137; *P*<.001) were associated with subsequent gait speed. Simultaneously, baseline gait speed was also associated with subsequent global cognition (β=.056, 95% CI 0.024-0.087; *P*=.001), mental intactness (β=.039, 95% CI 0.008-0.069; *P*=.01), and episodic memory (β=.057, 95% CI 0.023-0.092; *P*=.001). The comparison of standardized cross-lagged coefficients suggested that the effect size of baseline global cognition on subsequent gait speed was significantly larger than the reverse effect (*χ*_1_^2^=6.50, *P* for difference=.01). However, the effects of both mental intactness and episodic memory on subsequent gait speed were not significantly stronger than those of the reverse pathway (*χ*_1_^2^=3.33, *P* for difference=.07 and *χ*_1_^2^=3.21, *P* for difference=.07). Linear mixed-effects analyses further supported these bidirectional relationships, revealing that lower baseline cognitive scores predicted steeper declines in gait speed trajectory, and slower baseline gait speed predicted more declines in cognitive trajectory over time.

**Conclusions:**

There is a longitudinal bidirectional association between usual gait speed and both global cognitive function and specific domains of mental intactness and episodic memory among Chinese older adults. Baseline global cognition is likely to have a stronger association with subsequent gait speed than the reverse pathway. This interlinkage is noteworthy and may have implications for public health. Maintaining normal cognitive function may be an important interventional strategy for mitigating age-related gait speed reduction.

## Introduction

Population aging is a global challenge, with China being one of Asia’s most rapidly aging countries. The seventh population census in China reported that adults aged 60 years or older account for 18.70% of the total population, meaning that the prevalence of age-related diseases in China is set to increase dramatically [1-3]. Cognitive function is a vital dimension of healthy aging [4,5]. Cognitive function declines with age, which subsequently leads to many age-related diseases. Gait speed, an indicator that is easier to measure than other parameters, has been used as a simple, reliable, and sensitive indicator to assess and reflect the functional capacity and overall health [6,7]. Diminished gait speed is considered a strong predictor of the onset of negative outcomes in older adults [7]. Interestingly, declines in cognitive and gait speed often occur concurrently in older adults and are often related [8,9]. Moreover, these 2 common geriatric symptoms are associated with future adverse events, including falls, sarcopenia, disability, dementia, and even death, leading to a heavy burden on public health [10-13]. Therefore, cognitive function and gait speed have drawn widespread attention from public health and clinical researchers as health-related factors leading to age-related diseases.

Previous cross-sectional studies have reported an association between cognitive function and gait speed in older adults [14-19]. Several longitudinal studies have further investigated unidirectional temporal relationships, but the results have been inconsistent. For instance, longitudinal data from the Tasmanian Study of Cognition and Gait [18] revealed that cognitive decline predicted decreases in gait speed. A multicenter randomized controlled trial from European countries also showed that poorer baseline cognition was linked to an incident slow gait [20]. Evidence from older American adults further supported this unidirectional relationship [21,22]. However, a recent prospective study in Sweden suggested that slow gait speed predicts poor cognition [23]. Similar results were also found in older Australian and Japanese adults [24-26]. Furthermore, the currently available studies examining the longitudinal bidirectional association between cognition and gait speed have been conducted mainly in Western countries. Among these, 2 studies from the United States and 1 from the United Kingdom reported that cognition and gait speed were associated with each other over time [9,27,28]. While 2 longitudinal American studies also reported a reciprocal relationship, one [29] found that slower gait speed may be the driving factor in older men and women, whereas the other [30] found that cognitive decline was more likely to predict slower gait speed in older women. However, findings from a sample of 1478 older adults in the Mayo Clinic Study of Aging suggested that slow gait speed is likely a precursor to later cognitive decline, but not vice versa [31].

In summary, it appears that there is a relationship between cognitive function and gait speed, but the temporal sequence remains unclear. Moreover, due to methodological limitations (eg, linear mixed-effects models) [28,30,31], most previous studies could only unidirectionally examine the effect of gait speed on cognition or the effect of cognition on gait speed, but failed to simultaneously examine bidirectional associations between the 2 factors or examine temporal precedence [29,32]. Finally, relevant studies were primarily conducted among Western people [9,27-31], which differed greatly from China in terms of socioeconomic development, culture, and nutritional intake. To the best of our knowledge, the longitudinal reciprocal association between cognition and gait speed and its temporal sequence have not been examined in China.

Therefore, to fill these research gaps, this study aims to investigate the longitudinal bidirectional relationship between cognition and gait speed using a cross-lagged panel design, and determine the possible temporal sequence of the changes in the 2 factors in older Chinese community-dwelling adults. The clarity of the temporal sequence of cognitive decline and decreased gait speed might help identify the underlying causal predominance and early interventions for preventing age-related diseases.

## Methods

### Participants

**Figure 1:**
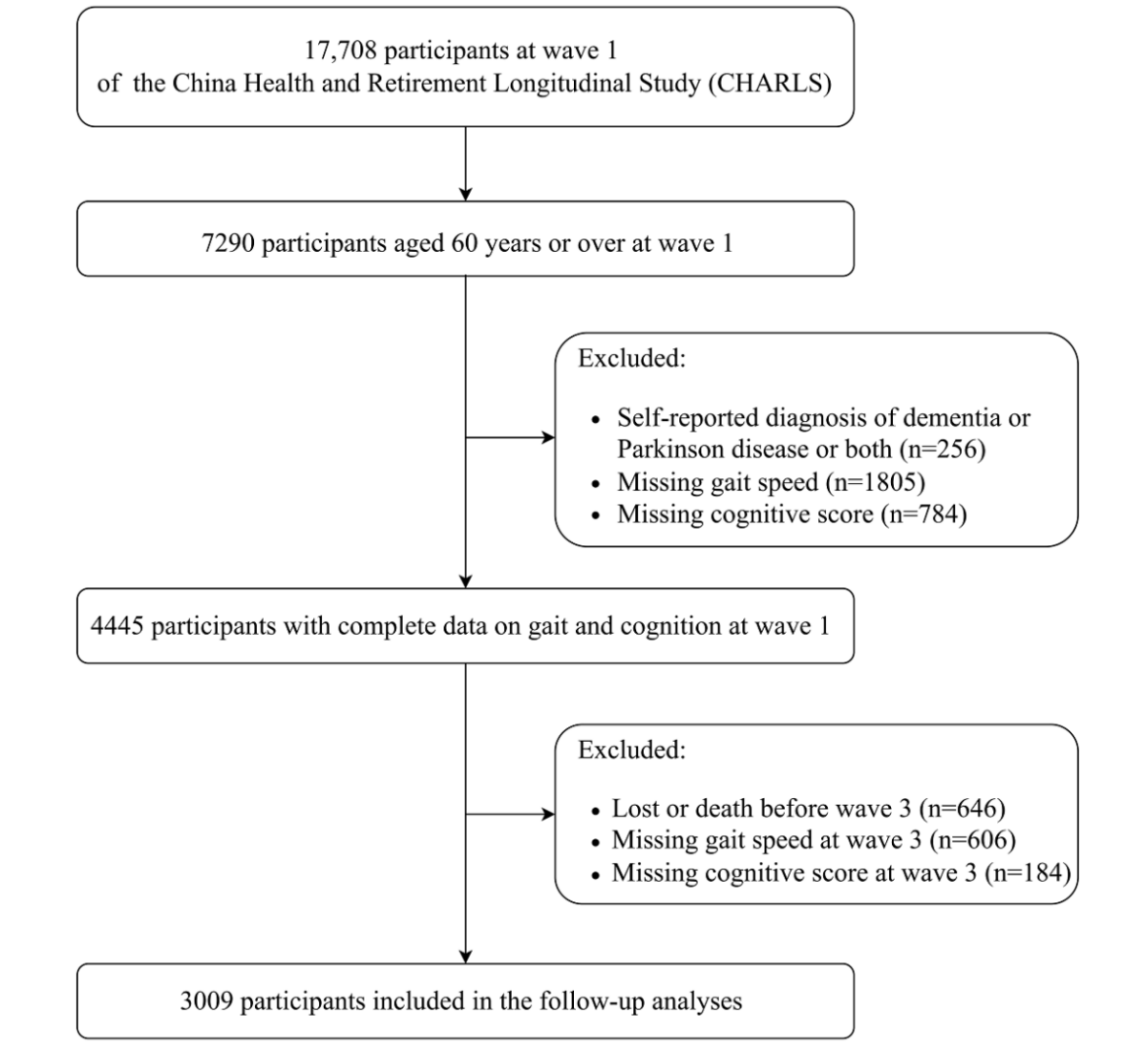
Flowchart of participants' selection.

Data were obtained from the China Health and Retirement Longitudinal Study (CHARLS), a nationwide population-based prospective cohort survey of adults aged 45 years or older. Further details on the study design and sampling strategies of the CHARLS have been described previously [33]. Briefly, in the baseline survey, a multistage stratified probability-proportionate-to-size sampling was adopted, and 17,708 adults from 450 villages or urban communities in 150 counties across 28 provinces in China were recruited. The baseline survey was implemented during the 2011-2012 period (wave 1), with subsequent waves completed approximately every 2 years, and data from wave 3 were collected from 2015 to 2016. This study included 7290 participants aged 60 years or older at baseline. Among these, participants were excluded due to a self-reported diagnosis of dementia or Parkinson disease or both at wave 1 (n=256), loss or death before wave 3 (n=646), or not having finished the gait speed test (n=1805 at wave 1 and 606 at wave 3) or cognitive tests (n=784 at wave 1 and 184 at wave 3). Ultimately, 3009 individuals were included in this study (Figure 1).

### Assessment of Cognitive Function

Two well-established composite measures were used to assess global cognitive function: mental intactness and episodic memory, which have been validated in Chinese people [34]. These metrics are similar to those used in the American Health and Retirement Study and in previous studies [35,36]. Mental intactness was based on components from the Telephone Interview of Cognitive Status, which described time orientation (naming the month, day, year, week, and season), numerical ability (serial subtraction of 7 from 100, 5 times), and visual and spatial abilities (redrawing task of 2 overlapping pentagons) [36,37]. Mental intactness scores were equal to the sum of the correct answers, with numbers ranging from 0 to 11. Episodic memory was based on immediate and delayed word recall tests. After the interviewer randomly read a list of 10 Chinese nouns, the respondents were asked to memorize as many words as they could immediately (immediate recall) and several minutes later (delayed recall). Episodic memory scores were the sum of correctly repeated words, ranging from 0 to 20. Global cognition scores were the aggregate of mental intactness and episodic memory scores ranging from 0 to 31, with higher scores indicating better cognitive performance.

### Assessment of Gait Speed

Gait speed was computed over 2 straight 2.5-m path walking courses. Those who had difficulty stopping walking (eg, recent surgery, injury, or health problems) were excluded from this test. Eligible older participants were instructed to walk 2.5 m along a noncarpeted walking course (there and back) at their usual speed. Timing began when one of the participants’ feet crossed the start line and landed completely, and ended when the participant crossed the finish line completely. The average velocity (m/s; accurate to 2 decimal places) was calculated, with higher scores indicating faster speeds.

### Covariates

The following baseline measurements were included as potential covariates: age, gender, residence region, marital status, educational level, self-reported health, current smoking and drinking status, self-reported visual and hearing impairments, depressive symptoms, restrictions on activities of daily living (ADL), and BMI. Information on chronic diseases included hypertension, diabetes mellitus, dyslipidemia, heart disease, stroke, cancer, lung disease, arthritis, kidney disease, digestive disease, and asthma. The number of diseases was used as a covariate. Restriction was considered as 1 or more dependency in ADL, including dressing, bathing, eating, getting into/out of bed, and using the toilet [38]. Depressive symptoms were measured using 10 items from the Center for Epidemiologic Studies Depression Scale, and a score of 10 or more was defined as positive [39].

### Statistical Analysis

**Figure 2:**
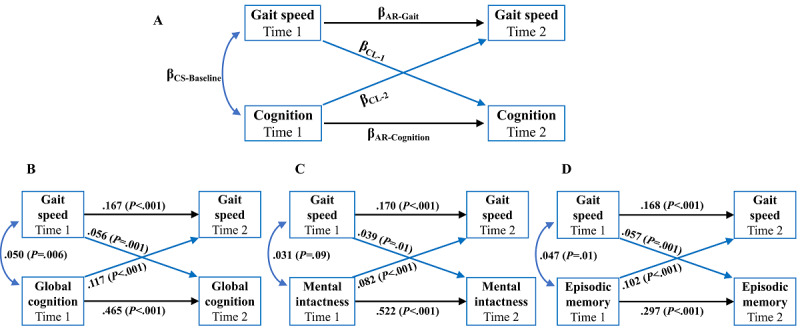
Cross-lagged panel models applied to assess gait speed and cognition among Chinese older adults. (A) The general modeling strategy used for the cross-lagged panel models. (B) The cross-lagged panel model where gait speed was associated with global cognition. (C) The cross-lagged panel model where gait speed was associated with mental intactness. (D) The cross-lagged panel model where gait speed was associated with episodic memory. Numeric values are standardized structural regression coefficients. AR: autoregressive; CL: cross-lagged; CS: cross sectional.

Continuous variables are presented as means and SDs and categorical variables are shown as numbers (proportions). Cross-lagged path models were first performed to examine the possible longitudinal reciprocal association between cognition and gait speed measured at 2 time points 4 years apart [32,40]. Figure 2A demonstrates this study’s general modeling strategy, including 5 paths and their corresponding coefficients. Cross-lagged paths concurrently include 2 cross-lagged coefficients, β_CL-1_ and β_CL-2_. β_CL-1_ represents the effect of gait speed at time 1 on cognitive scores at time 2, whereas β_CL-2_ implies the regression of cognitive scores at time 1 on gait speed at time 2. The temporal sequence was determined by comparing the estimated standardized cross-lagged coefficients [40]. Cross-sectional paths between gait speed and cognitive scores were also modeled, and the coefficient β_CS-Baseline_ at time 1 represented the baseline correlation between gait speed and cognitive scores. Finally, 2 autoregressive coefficients, β_AR-Gait_ and β_AR-Cognition_, were obtained from the paths between times 1 and 2 for gait speed and cognitive scores, respectively, accounting for with-person stability in each measure from the baseline to the subsequent time.

Before the cross-lagged path analysis, the baseline and subsequent values of gait speed and cognitive scores were adjusted for a series of covariates by regression residual analyses, and then standardized with Z-transformation (mean 0, SD 1). Standardized coefficients were reported from the 4 models. A crude model and 3 multivariable-adjusted models were built: model 1 was built without any adjustments; model 2 was adjusted for age and sex; age, sex, residence, marital status, educational level, and smoking and drinking status were further controlled in model 3; and finally, model 4 was comprehensively adjusted, including the covariates in model 3 plus self-reporting of health scores, number of diseases, depressive symptoms, visual and hearing impairments, restriction on ADL, and BMI. A comparative fit index (CFI) >0.90 and standardized root-mean-square residual (SRMR) <0.05 were used to suggest the good fitness of the models [41]. Linear mixed-effects models were further implemented to visualize the longitudinal associations between cognition and gait speed with aging.

Several sensitivity analyses were performed. First, we controlled for the specific chronic diseases (11 diseases in total) instead of the number of diseases in model 4. Second, in addition to the covariates in model 4, we further adjusted for living environmental factors to control for their potential confounding, including the type of accommodation (multistory or 1 story), water sources (running water or no running water), room temperature (comfortable or uncomfortable), and household air pollution (clean fuels for both heating and cooking, clean fuel for heating or cooking, solid fuels for both heating and cooking) [42]. Finally, we excluded individuals with extremely low gait speed or global cognitive function scores or both at baseline (≤mean – 2 SD) to reduce the potential influence of the reverse temporal sequence. All analyses in this study were conducted in Stata 16.0 (StataCorp) and *P*≤.05 (2-tailed) was considered statistically significant.

### Ethical Considerations

The Biomedical Ethics Review Committee of Peking University approved the ethical application for the collection of human participant data for the CHARLS (IRB00001052-11015). The study data were anonymous. All the respondents provided written informed consent before the survey.

## Results

Ultimately, a national sample of 3009 adults aged 60 years or older was included, around half of whom were female (1422/3009, 47.26%). The mean age of the participants at baseline was 66.4 (SD 5.4) years. The baseline characteristics of the participants are summarized in Table 1.

Table 2 presents the results of the cross-lagged panel models. In models 1, 2, and 3, the standardized structural regression coefficients for all paths were strongly significant (Table 2). Fully adjusting for potential confounders diminished the effect size of all associations, but mostly remained statistically significant (Table 2); therefore, we mainly presented results from model 4. Moreover, autoregressive paths in all models for gait speed and cognition were highly remarkable, with coefficients not close to 0, capturing relative stability and influence from the baseline to the subsequent time point [40].

Figure 2B illustrates the cross-lagged path analysis of the gait speed and global cognitive scores in model 4. Cross sectionally, gait speed was positively associated with global cognitive scores (β=.05, 95% CI 0.014-0.086; *P*=.006). In addition, there was a significant cross-lagged pathway, and the standardized path coefficient from global cognitive scores at time 1 to gait speed at time 2 (β=.117, 95% CI 0.082-0.152; *P*<.001) was significantly stronger than that from gait speed at time 1 to global cognitive scores at time 2 (β=.056, 95% CI 0.024-0.087; *P*=.001; *χ*_1_^2^=6.50, *P* for difference=.01). Fitting indicators (CFI=0.952 and SRMR=0.034) suggested a good model fit based on the criteria of CFI>0.90 and SRMR<0.05 (Table 2).

Figure 2C shows the cross-lagged model of gait speed and mental intactness scores in model 4. At baseline, the correlation was not significant (β=.031, 95% CI 0.005-0.067; *P*=.09). However, there was a positive association between mental intactness scores at time 1 and gait speed at time 2 (β=.082, 95% CI 0.047-0.118; *P*<.001), and gait speed at time 1 was also associated with mental intactness scores at time 2 (β=.039, 95% CI 0.008-0.069; *P*=.01). The standardized path coefficient from mental intactness to gait speed was larger than that of the reverse pathway, but the difference was not significant (*χ*_1_^2^=3.33, *P* for difference=.07). The overall fit of this model was good (CFI=0.969 and SRMR=0.029; Table 2).

Figure 2D presents the results of gait speed and episodic memory scores in model 4. Gait speed was associated with episodic memory scores at baseline (β=.047, 95% CI 0.012-0.083; *P*=.01). For cross-lagged effects, episodic memory scores at time 1 were positively and significantly associated with gait speed at time 2 (β=.102, 95% CI 0.067-0.137; *P*<.001) and vice versa (β=.057, 95% CI 0.023-0.092; *P*=.001); however, the difference between the 2 standardized cross-lagged path coefficients was not significant (*χ*_1_^2^=3.21, *P* for difference=.07). The fitting parameters were CFI=0.928 and SRMR=0.031, indicating that the final model fits the data well (Table 2).

Multimedia Appendix 1 shows the baseline characteristics of living environmental factors of the study population. The first 2 sensitivity analyses showed similar bidirectional associations with model 4 (Multimedia Appendix 2). Specifically, when controlling for specific chronic diseases at baseline instead of the number of diseases or after additionally adjusting for living environmental factors, all cross-lagged path coefficients were still strongly significant (*P*<.05 in all cases; see Multimedia Appendix 2 for precise *P* values). In addition, after excluding 97 participants with extremely low gait speed or global cognitive scores or both, the association of gait speed with global cognition and episodic memory did not differ from model 4 (Multimedia Appendix 2). While the effect of baseline gait speed on subsequent mental intactness was slightly attenuated and became marginally significant (β=.030, 95% CI 0.002-0.061; *P*=.06), the reverse pathway still strongly held (β=.084, 95% CI 0.049-0.120; *P*<.001; Multimedia Appendix 2).

**Figure 3:**
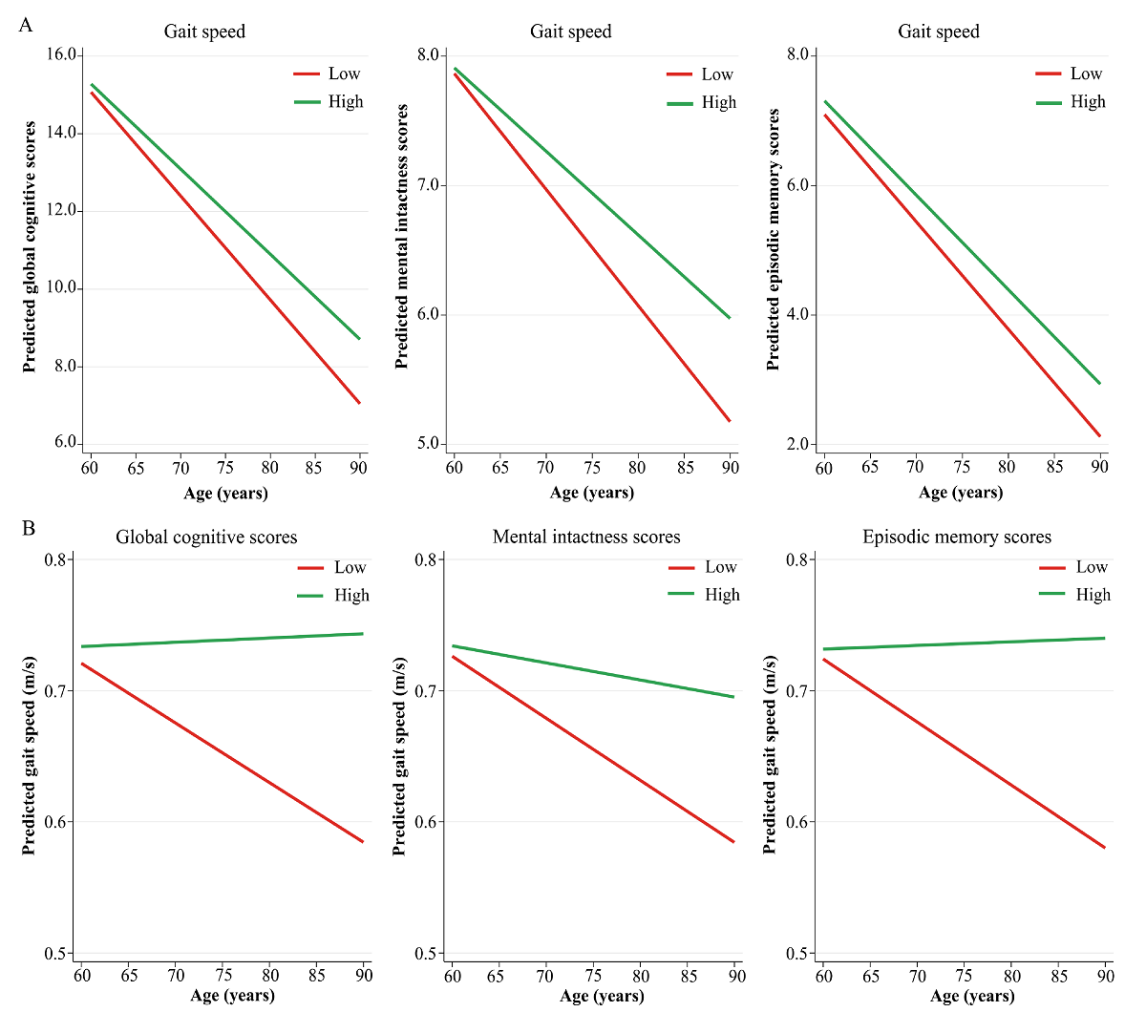
Trajectories of gait speed and cognition scores in a population-based study of Chinese older adults. (A) The predicted model estimates derived from linear mixed-effects models for global cognition, mental intactness, and episodic memory scores. The y-axis represents the predicted cognition scores based on model estimates, and separate lines indicate 1 SD above and below the mean gait speed (“high” and “low,” respectively). (B) Predicted model estimates derived from linear mixed-effects models of gait speed. The y-axis represents the predicted gait speed based on model estimates, and separate lines indicate 1 SD above and below the mean cognition scores (“high” and “low,” respectively).

Trajectories from Figure 3A further supported the temporal associations from cross-lagged path models, with slower baseline gait speed (1 SD below the mean baseline speed) predicting faster decline rates in global cognitive, mental intactness, and episodic memory scores over time. In Figure 3B, the trajectories were also consistent with the current results; older adults with lower (1 SD below the mean baseline score) baseline cognitive scores appeared to have steeper declines in gait speed than those with higher scores across the follow-up period.

**Table 1:** Baseline characteristics of the study population (N=3009).

Characteristic		Values
Age (years), mean (SD)		66.4 (5.4)
Female, n (%)		1422 (47.26)
Rural residence, n (%)		2020 (67.13)
Married, n (%)		2412 (80.16)
**Educational level, n (%)**		
	No formal education	1609 (53.47)
	Primary school	853 (28.35)
	Middle school	390 (12.96)
	High school or above	157 (5.22)
Current smoker, n (%)		977 (32.47)
Current drinker, n (%)		969 (32.20)
Self-report of health score, mean (SD)		3.1 (0.9)
**History of diseases, n (%)**		
	Hypertension	968 (32.17)
	Diabetes	202 (6.71)
	Dyslipidemia	303 (10.07)
	Heart diseases	444 (14.76)
	Stroke	69 (2.29)
	Cancer	23 (0.76)
	Lung disease	394 (13.09)
	Arthritis	1082 (35.96)
	Kidney disease	178 (5.92)
	Digestive disease	682 (22.67)
	Asthma	200 (6.65)
Number of diseases, mean (SD)		1.6 (1.5)
**Comorbidity, n (%)**		
	0	809 (26.89)
	1	875 (29.08)
	≥2	1325 (44.03)
Visual impairment, n (%)		252 (8.37)
Hearing impairment, n (%)		351 (11.67)
Depressive symptoms, n (%)		1165 (38.72)
Restriction on ADL^a^, n (%)		529 (17.58)
BMI (kg/m^2^), mean (SD)		23.0 (3.7)
**Weight status, n (%)**		
	Underweight	240 (7.98)
	Normal	1353 (44.97)
	Overweight	600 (19.94)
	Obesity	789 (26.22)
Gait speed (m/s), mean (SD)		0.6 (0.2)
Global cognitive scores, mean (SD)		13.9 (5.1)
Mental intactness scores, mean (SD)		7.4 (3.0)
Episodic memory scores, mean (SD)		6.5 (3.1)

^a^ADL: activities of daily living.

**Table 2:** Cross-lagged panel model results for gait speed and cognition scores in a population-based study of older adults.^a^

Gait and cognition results			Gait→cognition^b^		Cognition→gait^b^		Cross sectional^b^		Autoregressive^b^					Fit indices						
			β_CL-1_^c^		β_CL-2_^d^		β_CS-Baseline_^e^		β_AR-Gait_^f^		β_AR-Cognition_^g^		CFI^h^			TLI^i^		RMSEA^j^		SRMR^k^
**Global cognitive scores**																				
	Model 1^l^	.098^m^		.244^m^		.175^m^		.227^m^		.630^m^		0.944			0.719		0.204		0.045	
	Model 2^n^	.066^m^		.175^m^		.112^m^		.187^m^		.594^m^		0.963			0.817		0.142		0.034	
	Model 3^o^	.060^m^		.124^m^		.077^m^		.183^m^		.479^m^		0.956			0.782		0.122		0.033	
	Model 4^p^	.056^q^		.117^m^		.050^q^		.167^m^		.465^m^		0.952			0.758		0.122		0.034	
**Mental intactness scores**																				
	Model 1^l^	.076^m^		.216^m^		.165^m^		.235^m^		.630^m^		0.962			0.812		0.178		0.037	
	Model 2^n^	.049^m^		.147^m^		.094^m^		.193^m^		.644^m^		0.975			0.874		0.126		0.029	
	Model 3^o^	.042^q^		.091^m^		.055^q^		.188^m^		.535^m^		0.972			0.862		0.105		0.028	
	Model 4^p^	.039^r^		.082^m^		.031		.170^m^		.522^m^		0.969			0.846		0.107		0.029	
**Episodic memory scores**																				
	Model 1^l^	.118^m^		.186^m^		.127^m^		.246^m^		.404^m^		0.894			0.470		0.195		0.052	
	Model 2^n^	.081^m^		.143^m^		.091^m^		.194^m^		.376^m^		0.927			0.634		0.135		0.038	
	Model 3^o^	.065^m^		.107^m^		.067^m^		.185^m^		.305^m^		0.927			0.634		0.135		0.038	
	Model 4^p^	.057^q^		.102^m^		.047^r^		.168^m^		.297^m^		0.928			0.638		0.104		0.031	

^a^See Figure 2 for a cross-lagged panel model diagram as a reference.

^b^Standardized structural regression coefficients.

^c^β_CL-1_ is cross-lagged path 1, where gait speed at time 1 predicts cognition scores at time 2.

^d^β_CL-2_ is cross-lagged path 2, where cognition scores at time 1 predict gait speed at time 2.

^e^β_CS-Baseline_ is the cross-sectional association between gait speed and cognition scores within time 1.

^f^β_AR-Gait_ is the autoregressive coefﬁcient for gait speed.

^g^β_AR-Cognition_ is the autoregressive coefﬁcient for cognition scores.

^h^CFI: comparative ﬁt index.

^i^TLI: Tucker-Lewis Index.

^j^RMSEA: root-mean-square error of approximation.

^k^SRMR: standardized root-mean-square residual.

^l^Model 1 was a crude model.

^m^*P*<.001.

^n^Model 2 was adjusted for age and sex.

^o^Model 3 was adjusted for age, sex, residence, marital status, educational level, smoking, and drinking status.

^p^Model 4 was adjusted for covariates in model 3 plus self-reported health score, number of diseases, depressive symptoms, visual and hearing impairment, restriction on activities of daily living, and BMI.

^q^*P*<.01.

^r^*P*<.05.

## Discussion

### Principal Findings

During the 4-year follow-up among Chinese older adults, there was a longitudinal bidirectional association between global cognitive function, mental intactness, episodic memory, and gait speed after accounting for baseline levels and possible covariates. In addition, the comparison of standardized cross-lagged coefficients suggested that the temporal association from baseline poor global cognition to subsequent slow gait speed may be stronger than vice versa. Trajectories derived from linear mixed-effects models added further evidence on the dynamic associations, with poorer baseline cognition and slower gait speed predicting faster decreases in gait speed and declines in cognition, respectively.

Similar to previous cross-sectional studies [8,16,17], we also found that gait speed was reciprocally and positively associated with cognitive function at baseline among older adults. However, longitudinal studies may provide stronger evidence in the temporal direction. Two previous longitudinal studies, both using linear mixed-effects models, have shown different results, with one [28] reporting a bidirectional association between cognition and gait speed among older individuals in the United Kingdom, and the other suggesting that faster initial gait speed was associated with less later cognitive decline among older American adults, but not vice versa [31]. This inconsistency may be attributable to differences in the mean age, sample size, and heterogeneity of walking speed measures. Nevertheless, owing to a unidirectional preset, this statistical method cannot simultaneously examine bidirectional associations of 2 time-varying factors or temporal precedence [29,32]. Therefore, we fitted cross-lagged path models based on structural equation models and visualized temporal association using linear mixed-effects models, as suggested by a previous study in this area [29].

Our findings provided new insights into the nature of age-related changes in gait speed and cognition. To the best of our knowledge, only a few studies have elucidated the longitudinal reciprocal association between cognition and gait speed in older adults. Two such studies, also based on structural equation models, have also found bidirectional relationships between poor cognition and slow gait speed among older American people [9,27]. Specifically, 1 study included 412 participants aged 60 or older, finding that executive function, but not memory, predicted subsequent usual gait speed, and vice versa [9]. However, another study conducted in women aged 70-79 years reported a mutual association between gait speed and global cognitive function and particular memory. In our study, there was a bidirectional relationship between global cognition and gait speed and between specific domains (mental intactness and episodic memory) and gait speed. The mental status in the CHARLS describes time orientation, numerical ability, and visual and spatial abilities, and is similar to executive function [43]. Compared with these 2 studies, the sample size in our study was relatively larger. Our study had a 4-year follow-up duration that was shorter than the 9- and 6-year follow-up periods. Different cognitive tests on specific domains and discrepancies in the gender of the participants may also lead to this inconsistency. Therefore, more similar studies in older Chinese individuals are needed to verify whether the results hold across a longer follow-up interval or in sex-specific older adults. Another notable study among older American adults reported that early gait speed decline may be a precursor to later global cognitive decline and that the reciprocal association was weaker [29]. Although this study also revealed reciprocal relationships between the 2 factors, its temporal sequence contradicts ours. In our analysis, the standardized cross-lagged coefficient of baseline global cognition on follow-up gait speed was nearly 2 times that of baseline gait speed on subsequent cognition, indicating that global cognitive decline might be the cause of decreased gait speed than its result. The reasons for this discrepancy may be the difference in participants’ baseline average age and the length of the follow-up period, given that both cognition and gait speed decline over time in older adults [8]. Our study participants were over 60 years old, while the older adults were 70-79 years in their study. Moreover, their design had a longer follow-up time of 9 years than our 4-year interval. Thus, the current temporal sequence of cognitive decline to gait slowing requires further evidence, especially in older Chinese individuals.

Consistent with prior unidirectional longitudinal studies [18,21,22,44], our results suggest that poor baseline cognition predicted gait speed decline after 4 years, irrespective of the exclusion of those with very low baseline gait speed and global cognition. Moreover, poor specific domains of cognition, including mental intactness and episodic memory, are predictive of decreased gait speed, which supports the results from previous studies [28,45]. A small pilot randomized controlled trial showed that improvements in domains of cognition, including executive function, attention, visuospatial skills, and processing speed, were associated with increases in walking speed [45]. Memory has also been reported to be positively and longitudinally associated with gait speed in older adults, supporting our results [28].

Although we found a stronger directionality of early cognitive change in subsequent gait speed change than the opposite pathway, we do not discount the importance of early gait change. As with prior results [24,26], although weak, we again demonstrated the predictive role of baseline faster gait speed in later cognition. A recent study also revealed that decreased gait speed precedes global cognitive decline during a longer follow-up duration than ours [23]. Evidence from a prospective study in 70-89-year older adults has also shown results similar to ours [31], with baseline faster gait speed associated with better global cognition, executive function, and memory. In addition, both gait speed and cognitive function were important when assessing the risk of dementia in older adults [29,46].

Neuroimaging studies also support this reciprocal relationship. Age-related changes in gait and cognition have been reported to share common underlying structural alterations in the prefrontal and temporal lobes [47]. Furthermore, several mechanisms may explain the stronger effect of cognition on gait speed. First, adverse structural alterations in the brain, such as hippocampal atrophy, white matter, and an increase in white matter damage, are associated with decreasing gait speed in older adults [48]. Second, walking is a complex task that requires energy, balance, and coordination of multiple systems, including the nervous system, and thus disruption or deterioration of any system tends to decrease gait speed [6,49]. Finally, specific cognitive capacities may be vital for the initiation and maintenance of walking [22]. A functional magnetic resonance imaging study has shown that some areas with greater activation in the brain reflect increased cognitive monitoring of movements in older participants compared with that in younger people [50]. Overall, cognitive decline may precede or co-occur with gait speed decrease among older Chinese adults; therefore, early arrangements should be ensured to maintain both cognitive function and gait status to avoid further adverse outcomes. Meanwhile, cognitive decline might be a sensitive early marker of gait deceleration, indicating that cognition might be an essential factor to be considered along with gait speed to identify risks for gait speed decrease or mobility deﬁcits among older adults.

### Strengths and Limitations

This study has several strengths. Participants aged 60 years or older were from the CHARLS, which is a nationally representative, population-based prospective cohort in which cognitive functioning was assessed with well-validated measures. Relatively comprehensive information is available in the CHARLS, and related covariates were adjusted as much as possible in this study’s final model. In addition, this study expands upon cross-sectional and traditional longitudinal analyses by performing cross-lagged panel models, which allowed for the capture of a temporal sequence by simultaneously evaluating the strengths of temporal bidirectional associations [32,40]. In addition, compared with similar studies [27,29], our study added linear mixed-effects models to visualize the temporal bidirectional relationships of the 2 factors. Finally, to our knowledge, this is the first study to examine the possible temporal sequence of changes in gait speed and cognition in older Chinese people, and our findings deserve further verification.

Several limitations also need to be considered. First, our analysis involved an approximately 4-year follow-up interval due to the limited availability of gait speed information at the later wave mark; therefore, it could not be determined whether this association continued over a longer period. Second, while the reciprocal associations between cognition and gait speed were statistically significant after accounting for a variety of potential confounding factors, there may still be some related but uncontrolled variables such as the apolipoprotein E4 (ApoE4) genotype. Slow walking speed in those with ApoE4 preceded lower memory scores [24], but unfortunately, these data are not available in the CHARLS. Based on a genomic study, the *e4* allele has a 6.9% frequency rate in the Chinese population; therefore, this genotype may have slightly affected our results [35]. Third, the older participants were allowed walking aids for this study, which may obscure the true association between gait and cognition to some extent. As one recent study has shown, the cross-sectional correlation between cognition and gait may be attenuated when considering the interaction of walking-aid usage [8]. Eventually, gait speed was measured at a usual pace, with this study solely focused on this walking condition; therefore, we were unable to determine whether the relationships we reported agree with the results obtained under other walking conditions, such as maximum walking speed, and this requires further research to identify.

### Conclusions

This study demonstrates a longitudinal reciprocal association of gait speed with global cognitive function, mental intactness, and episodic memory among Chinese older adults. Cross-lagged path coefficients suggest that baseline global cognition is likely to have a stronger association with subsequent gait speed than the reverse pathway. These findings further reinforce the importance of promoting both cognition and gait conditions early, and provide ideas for improving the clarity of the causal direction of cognition and gait speed changes. Maintaining normal cognitive function may be an important interventional strategy for mitigating age-related gait speed decrease.

## Appendix

Multimedia Appendix 1 Baseline characteristics (living environmental factors) of the study population.

Multimedia Appendix 2 Cross-lagged panel model results for gait speed and cognition scores in a population-based study of older adults (sensitivity analyses).

## Abbreviations

ADL: activities of daily living
CFI: comparative fit index
CHARLS: China Health and Retirement Longitudinal Study
SRMR: standardized root-mean-square residual

## Data Availability

The China Health and Retirement Longitudinal Study (CHARLS) data set is publicly available at [52]. The data analyzed in our study can be obtained from the corresponding author upon request.
