# Supplementary material for: The Bidirectional Association Between Cognitive Function and Gait Speed in Chinese Older Adults: Longitudinal Observational Study
Source: JMIR Public Health Surveill. 2023 Mar 14;9:e44274. doi: 10.2196/44274 (PMC10131755; doi:10.2196/44274)
Supplement: Multimedia Appendix 1 [file publichealth_v9i1e44274_app1.docx]

| **Multimedia Appendix 1.** Baseline characteristics (living environmental factors) of the study population. | |
| --- | --- |
| **Characteristic** | **Value** |
| Type of accommodation, n (%) |  |
| Multi-story | 997 (33.2) |
| One-story | 2009 (66.8) |
| Water sources, n (%) |  |
| Running water | 1777 (59.2) |
| No running water | 1227 (40.9) |
| Room temperature, n (%) |  |
| Comfortable | 2512 (83.6) |
| Uncomfortable | 492 (16.4) |
| Household air pollution, n (%) |  |
| Both clean fuels | 499 (20.9) |
| Clean fuel or solid fuel | 534 (22.4) |
| Both solid fuels | 1356 (56.8) |
| *Note*: Values of polytomous variables may not sum to 100% due to rounding. | |
