# Supplementary material for: The Bidirectional Association Between Cognitive Function and Gait Speed in Chinese Older Adults: Longitudinal Observational Study
Source: JMIR Public Health Surveill. 2023 Mar 14;9:e44274. doi: 10.2196/44274 (PMC10131755; doi:10.2196/44274)
Supplement: Multimedia Appendix 2 [file publichealth_v9i1e44274_app2.docx]

| **Multimedia Appendix 2.** Cross-lagged panel model results for gait speed and cognition scores in a population-based study of older adults (sensitivity analyses).^a^ | | | | | | | | | | | | | |
| --- | --- | --- | --- | --- | --- | --- | --- | --- | --- | --- | --- | --- | --- |
|  | **Standardized Structural Regression Coefﬁcients** | | | | | | | |  |  |  |  |  |
|  | **Gait → Cognition** |  | **Cognition → Gait** |  | **Cross-Sectional** |  | **Autoregressive** | |  | **Fit Indices** | | | |
| **Gait and Cognition** | **β_CL-1_^b^** |  | **β_CL-2_^c^** |  | **β_CS-Baseline_^d^** |  | **β_AR-Gait_^e^** | **β_AR-Cognition_^f^** |  | **CFI^g^** | **TLI^h^** | **RMSEA^i^** | **SRMR^j^** |
| **1. Controlling for specific chronic diseases at baseline instead of the number of diseases** | | | | | | | | | | | | | |
| Global cognitive scores | .057^l^ |  | .117^k^ |  | .049^l^ |  | .173^k^ | .467^k^ |  | 0.953 | 0.764 | 0.122 | 0.033 |
| Mental intactness scores | .040^m^ |  | .084^k^ |  | .032 |  | .176^k^ | .522^k^ |  | 0.969 | 0.843 | 0.108 | 0.029 |
| Episodic memory scores | .059^l^ |  | .102^k^ |  | .046^m^ |  | .174^k^ | .299^k^ |  | 0.932 | 0.658 | 0.102 | 0.030 |
| **2. Additionally adjustments for living environmental factors** | | | | | | | | | | | | | |
| Global cognitive scores | .057^l^ |  | .122^k^ |  | .055*^l^* |  | .199^k^ | .462^k^ |  | 0.936 | 0.680 | 0.144 | 0.039 |
| Mental intactness scores | .038^m^ |  | .094^k^ |  | .051^m^ |  | .201^k^ | .526^k^ |  | 0.962 | 0.810 | 0.122 | 0.032 |
| Episodic memory scores | .058^l^ |  | .100^k^ |  | .038 |  | .202^k^ | .302^k^ |  | 0.911 | 0.556 | 0.122 | 0.036 |
| **3. Excluding those with extremely low gait speed and/or global cognitive scores** | | | | | | | | | | | | | |
| Global cognitive scores | .054^l^ |  | .121^k^ |  | .045^m^ |  | .164^k^ | .453^k^ |  | 0.951 | 0.753 | 0.120 | 0.033 |
| Mental intactness scores | .030 |  | .084^k^ |  | .025 |  | .167^k^ | .512^k^ |  | 0.971 | 0.853 | 0.102 | 0.027 |
| Episodic memory scores | .062^l^ |  | .103^k^ |  | .043^m^ |  | .164^k^ | .292^k^ |  | 0.924 | 0.619 | 0.105 | 0.031 |

^a^See Figure 2 for the cross-lagged panel model diagram as reference.

^b^β_CL-1_ is cross-lagged path 1, where gait speed at time 1 predict cognition scores at time 2;

^c^β_CL-2_ is cross-lagged path 2, where cognition scores at time 1 predict gait speed at time 2;

^d^β_CS-Baseline_ is the cross-sectional association between gait speed and cognition scores within time 1;

^e^β_AR-Gait_ is the autoregressive coefﬁcient for the gait speed;

^f^β_AR-Cognition_ is the autoregressive coefﬁcient for the cognition scores.

^g^CFI = comparative ﬁt index;

^h^TLI = Tucker-Lewis Index;

^i^RMSEA = root mean square error of approximation;

^j^SRMR = standardized root mean square residual.

^k^*P* <.001; ^l^*P* <.01; ^m^*P* <.05.
